# Influence of Mind upon the Physical System

**Published:** 1856-10

**Authors:** T. C. Patterson


### IOWA MEDICAL JOURNAL.

VOL. III. KEOKUK, IOWA, SEPT. AND OCT., 1856. NO. 5.

### ORIGINAL COMMUNICATIONS.

### INFLUENCE OF MIND UPON THE PHYSICAL SYSTEM. A PAPER READ BEFORE THE KNOX COUNTY MEDICAL SOCIETY, ILLINOIS,

BY T. C. PATTERSON, M. D.

I esteem it a privilege to be permitted to call your attention to a subject, which, to my mind, possesses peculiar interest, viz: the mysterious and controlling influence that the mind of man possesses over the animal or physical system. This subject is enshrouded in such deep mystery, that I dare not attempt to fathom its depths or scale its heights. Man, to himself, is a mystery, and in this respect, like the mathematical investigations of the durability of eternity, the more we reflect on it, the further we are from the end. We have a history of man in the good Book—God made man and breathed into his nostrils the breath of life, and he became a living being. We learn from the same ancient and revered record, that man possesses a soul and body —that the soul is that ever-living, never-dying, immortal part, which cannot be seen, handled, or felt. It is the thinking part which is capable of solving great problems, measuring the distance of earth from Heaven, and numbering the planets, contemplating, with amazing interest and delight, their magnitude, and calculating the revolutions of the earth we live upon. It is that part that turns around, as it were, and scrutinizes its connection with a lump of clay, with which it is encumbered, and often desires to be freed from it; but lingers longer and sighs in discontentment until the unseen angel or messenger of death releases it, when it takes its flight to Heaven, or is justly cast down to hell. Here the christian philosopher, or physician of the soul, takes one branch of this subject and goes on calculating the ponderous worth of, or the magnitude ot the loss of a soul, &c. (A worthy subject for us all to meditate upon.) But as this does not seem to be the order of the day with us, and as it is our object and duty to try to keep soul and body together as long as possible, we return to inquire what control or influence the mind has over the body. And our hypothesis is that electricity is the connecting link between them, and through attraction and repulsion, the cause of all the phenomena visible in man. To those who have had neither time or inclination to ponder this grand subject, this may seem a startling and utterly untenable position, but a moment's reflection upon the thousand conflicting and unsatisfactory theories which have, in succession, gained and lost reputation in the medical world, in regard to the connection of mind with matter, its influence in and also concerning the primary cause of all disease, will convince the candid mind that it may be founded in truth. The Atheist denies the existence of spirit, and maintains that mind is the result of a peculiar organization of matter, while the Christian, on the other hand, insists that it is an immaterial essence. It may be well before going further, to examine the grounds on which the two doctrines, so diametrically opposite in their nature and tendency, are based. The Atheist denies the existence of spirit, because he says he cannot gain the same evidence of its existence as he can of matter; he also lays down the principle of evidence that a man is not bound to believe that of which the senses cannot take cognisance, unless proven by mathematical demonstration. In regard to the first objection, I would make bold to affirm that there is fully as strong evidence of the existence of spirit as of inert matter. Take, for instance, electricity, a substance whose existence is unquestioned. We cannot see it; it writes its presence in the skies in blazing and eccentric tracks upon the dark bosom of the thunder cloud, but we do not see the subtile fluid itself, only the eflects of its inconceivably rapid motion; we do not hear it. The deafening thunder is but the rushing together of the air separated by this prime agent of the Deity's resistless path; we cannot smell nor can we taste it, but we can feel its effects and that within ourselves. Thus we perceive we cannot gain a knowledge of a known substance in nature by any of the senses, save through its effects. So with mind. We cannot see, hear, taste, or smell it, but every where we see its effects. We see them in the innate longings of man for a higher and nobler state of being, and the horror with which he shrinks from the idea that the higher and nobler portion of his being, capable of unlimited expansion, shall be cut off in its glorious career and quenched forever in the dark night of the grave. We see them in the stupendous achievements of thought, the result of its energy; it wars with and subdues all forms of matter, from the grossest to the most refined; it soars into unfathomable space and with unerring accuracy, numbers the globes that ceaselessly circle round the sun, measuring their distance and determining the time of their revolutions. Again, as we ascend in the scale of created matter, we find that those substances which possess the grosser attributes of weight, size, and visible shape, are controlled in all of their motions by those of an aeriform nature, and when we arrive at electricity, the only absolutely imponderable agent in nature, we find it lends to those beneath, all their powers. Through it the winds rush from their hiding places and lash the placid bosom of the ocean into remorseless fury. It invests the thunder with its awful sublimity; arms the hurricane with its terrific power; spreads, as a mantle over the Heavens, the surprisingly brilliant Northern light; wakes to activity the slumbering earthquake, and robes the sun in all its refulgent glory; yet as this inconceivably subtile, powerful, and ubiqutous agent, the highest in the chain of inert existence, and approximating nearest to motion, yet not possessing inherent motion, is capable of thought,—it follows as a natural and inevitable conclusion, that the substance before whose might even this subtile, imponderable agent bows in submission, must be spiritual, and the organ of all motion, power, and thought. In regard to the principle of evidence referred to, I would merely aver that when the Atheist can prove that of which all the bodily senses can take cognisance, viz: his bodily existence by any better argument than *existo ego sum*, it will be time enough for him to demand a mathematical demonstration of that which the bodily senses cannot take cognisance. The Christian affirms the immateriality of the mind, for precisely the same reason that the Atheist rejects its existence, the impossibility of gaining a knowledge of it as we do that of inert matter, and with just as little apparent reason. That which is immaterial is not composed of substance, is not substance, therefore cannot occupy space nor have shape, but looks very much like nothing at all. An immateriality is nothing, strictly speaking, but a blank, a nonentity. But this conclusion would be repulsive to the Christian mind. But again, what is mind? Were I to undertake to define, I would say it must be an inconceivably refined and subtile substance, as certainly a substance as iron or lead, but millions of times finer than electricity, which is itself one million times finer than air. How the imbodied mind staggers and faints in its endeavors to comprehend its own inconceivably subtile substance. If it be admitted, as proven, that mind is an independent existence, the next step to be taken is to enquire how it acts upon gross matter. It is certain that it does act upon gross matter, and it is equally certain that it cannot act upon it directly. This, we think, is capable of demonstration. If, for instance, the great nerve of the arm be severed, so as to cut off the communication from the brain, the mind may will the arm to rise but in vain, though the bones, muscles, and blood are all there. Let, however, the electric battery be applied, and the arm will rise by the application of the same. The phenomena of life may be produced in death, and the rigid corpse be made to open its glazed eyes, rear upright its ghastly form, heave its airless lungs, contract and relax the muscles of the limbs. By the application of the battery, the pimples commonly called goose pimples, may be produced, such as are produced by extraordinary emotions of the mind, as fear, joy, or sudden surprise. It may be also observed that these pimples are always at the roots of the small hairs which cover the body. The philosophy of this phenomena is evidently identical in both cases. Any sudden and powerful emotion of the mind calls in great force the electro-nervous fluid from the rest of the body, and concentrates it upon the brain, leaving the rest of the body highly negative. As the positive and negative forces of electricity rush together, the negative fluid of the body, in seeking the surrounding positive fluid of the atmosphere, meets, in its passage, a non-conductor in the small hairs which cover the body and raise pimples, and causes the hairs of the head to stand erect. Intense fright will cause the hairs of the head to stand erect and will draw so much of the electro-nervous fluid as to leave the limbs uterly powerless. Take a silk umbrella, for instance, closed and perfectly dry; rub it briskly for a minute or so, downwards, with the hands; stand its handle on the floor, balance it between the palms of the hands touching it near the top of the silk, then remove them, evenly and gently, for two or three inches away from it on each side; you may then move your hands backwards and forwards and the umberella will follow two or three inches out of its perpendicular, and again follow them back to its upright position. I would ask, is it not a fair conclusion, then, that the nervous fluid and electricity, possessing the same principles, being alike imponderable, invisible and intangible, and under the same circumstances, producing the same effects, are not one and the same fluid? By the same mode of reasoning only can we arrive at a knowledge of the identity of any two principles of the same substances, as wood or iron. If it be admitted that mind exists as an independent entity and controls the motions of the body through its agent, it follows that it must possess involuntary powers, corresponding with the involuntary motions of the body, and that the motions of the heart, lungs and the liver are equally under the control of the mind, with those of the limbs, for if the involuntary motions of the body were merely the result of organization, the voluntary powers of the mind could not possibly obtain any influence over them. But the will, we know, can exercise a powerful influence over all of the involuntary motions, and medical history informs us that this has been done to such an extent as to produce death. The journals of the medical offices of the British army inform us that Jugglers of that country have the singular power of suspending, for days, all involuntary motions and then causing them to resume. The voluntary powers of the mind may be lost by the distruction of the organs through which they act, and yet the involuntary continue their usual motions. In natural sleep, the voluntary powers of the mind are locked up in death or her twin sister—the organs through which the involuntary act are distroyed. In the next place, we will briefly notice the influence of mind, producing cures of disease. Upon no other than electrical principles can the influence of imagina tion be explained. Take, for instance, a case reported by Dr. Warren. A lady came and cousulted him in regard to the propriety of rubbing a cancer with which she was afflicted, with a dead man's hand. He at first thought of dissuading her, but reflecting upon the powers of immagination, he advised her to try it. She did so, in faith, and in a short time the cancer began to disappear, and finally vanished. The philosophy of this, we think, is plain. The nerves, blood vessels, the whole body of the tumor was nourished by the action of the fitty per cent of the Electro fluid under the control ot the involuntary powers of the mind. But by a strong effort of the will, a much larger proportion of the fifty per cent. under their control of the voluntary powers, was thrown to the part counteracting the efforts of the first, and finally causing the tumor to disappear. So in all diseases—the voluntary powers can throw a very large proportion of the fifty per cent. of Elictricity under their control upon the diseased part, and contribute, in a great measure, to heal it. We don't advocate that all diseases can be healed by mental impression; but many of the ills that flesh is heir to may be avoided, and when contracted, alleviated and often times cured by a knowledge of the principles which we are endeavoring to set forth in this essay. It is clear and indisputable, that the principles of health and disease are in man, and are one and the same. The vomiting principle is in the system or else why will a thought oftentimes produce the effect of a bilent emetic? The same may be said of the purgative, diaphoretic and diuretic. (This is observable among the lower animals,) and all can be made manifest in man by mental action. As all diseases are not, however, produced by mental action, so they cannot all be cured by mental impressions, exclusively. But the *modus operandi* of the curative principles is the same in all cases. A lady of delicate nervous organization imagines, for instance, while eating a dish of Strawberries, or other delicacy, that she has swallowed a fly; what is the consequence ? Precisely the same as if she had actually swallowed it. The action of the involuntary powers are directed to the stomach—the secretions are excited—the Electro-nervous fluid follows the moisture—collapse ensues, and vomiting is produced. This is presisely the philosophy of the action of any emetic. Medicines being given to excite, not produce, the vomiting principle or any other curative principle. The Physcian should, in all cases when strong reasons do not exist for not doing so, inform the patient of the effect disired to be produced, and do all in his power to excite the corresponding or co-operation of the energies of the mind to aid the action of the medicine and thus the more successfully control the disease. I have thus endeavored to state and explain as briefly as possible a principle in regard to the connection between mind and matter and the application of this principle in disease, to which subject too little attention has been paid by the profession. The subject is worthy of the calm and dispassionate consideration of scientific men. It is upon this principle that we can truly and satisfactorily account for marvelous yet absolute cures performed by the Homœopatheist the wide world over, and many of those who practice Homœopathically understand this well, and anchor their hope of success in operating upon the credulous, gaining their approbation and confidence. How is it that their efforts are crowned with success? There might be volumes of evidence produced in proof of this position. But I leave the subject, fully sensible of the many imperfections accompaning this effort and of my utter inability to do it justice. I therefore submit it to your consideration, and if you deem it of sufficent importance to give it a place in your Journal, you may do so, hopeing it may induce one more competent to investigate.

